# OX40 ligand expressed in glioblastoma modulates adaptive immunity depending on the microenvironment: a clue for successful immunotherapy

**DOI:** 10.1186/s12943-015-0307-3

**Published:** 2015-02-15

**Authors:** Ichiyo Shibahara, Ryuta Saito, Rong Zhang, Masashi Chonan, Takuhiro Shoji, Masayuki Kanamori, Yukihiko Sonoda, Toshihiro Kumabe, Masahiko Kanehira, Toshiaki Kikuchi, Takanori So, Takashi Watanabe, Hiroaki Takahashi, Erina Iwabuchi, Yuetsu Tanaka, Yukiko Shibahara, Hironobu Sasano, Naoto Ishii, Teiji Tominaga

**Affiliations:** Department of Neurosurgery, Tohoku University School of Medicine, 1-1 Seiryo-machi, Aoba-ku, Sendai, 980-8575 Miyagi Japan; Department of Neurosurgery, Kitasato University School of Medicine, Sagamihara, Kanagawa 252-0374 Japan; Department of Respiratory Oncology and Molecular Medicine, Tohoku University School of Medicine, Sendai, 980-8575 Miyagi Japan; Department of Immunology, Tohoku University School of Medicine, Sendai, 980-8575 Miyagi Japan; Department of Public Health, Tohoku University School of Medicine, Sendai, 980-8575 Miyagi Japan; Department of Pathology, Tohoku University School of Medicine, Sendai, 980-8575 Miyagi Japan; Department of Immunology, Graduate School of Medicine, University of the Ryukyus, Okinawa, 903-0215 Japan

**Keywords:** Glioblastoma, OX40, OX40 ligand, Immunotherapy, Hypoxia, Regulatory T cell

## Abstract

**Background:**

Glioblastoma is the most malignant human brain tumor and has a dismal prognosis; however, some patients show long-term survival. The interaction between the costimulatory molecule OX40 and its ligand OX40L generates key signals for T-cell activation. The augmentation of this interaction enhances antitumor immunity. In this present study, we explored whether OX40 signaling is responsible for antitumor adaptive immunity against glioblastoma and also established therapeutic antiglioma vaccination therapy.

**Methods:**

Tumor specimens were obtained from patients with primary glioblastoma (n = 110) and grade III glioma (n = 34). Quantitative polymerase chain reaction (PCR), flow cytometry, and immunohistochemistry were used to analyze OX40L expression in human glioblastoma specimens. Functional consequences of OX40 signaling were studied using glioblastoma cell lines, mouse models of glioma, and T cells isolated from human subjects and mice. Cytokine production assay with mouse regulatory T cells was conducted under hypoxic conditions (1.5% O_2_).

**Results:**

OX40L mRNA was expressed in glioblastoma specimens and higher levels were associated with prolonged progression-free survival of patients with glioblastoma, who had undergone gross total resection. In this regard, OX40L protein was expressed in A172 human glioblastoma cells and its expression was induced under hypoxia, which mimics the microenvironment of glioblastoma. Notably, human CD4 T cells were activated when cocultured in anti-CD3-coated plates with A172 cells expressing OX40L, as judged by the increased production of interferon-γ. To confirm the survival advantage of OX40L expression, we then used mouse glioma models. Mice bearing glioma cells forced to express OX40L did not die during the observed period after intracranial transplantation, whereas all mice bearing glioma cells lacking OX40L died. Such a survival benefit of OX40L was not detected in nude mice with an impaired immune system. Moreover, compared with systemic intraperitoneal injection, the subcutaneous injection of the OX40 agonist antibody together with glioma cell lysates elicited stronger antitumor immunity and prolonged the survival of mice bearing glioma or glioma-initiating cell-like cells. Finally, OX40 triggering activated regulatory T cells cultured under hypoxia led to the induction of the immunosuppressive cytokine IL10.

**Conclusion:**

Glioblastoma directs immunostimulation or immunosuppression through OX40 signaling, depending on its microenvironment.

**Electronic supplementary material:**

The online version of this article (doi:10.1186/s12943-015-0307-3) contains supplementary material, which is available to authorized users.

## Background

Glioblastoma, classified as grade IV glioma by the World Health Organization, is associated with a dismal prognosis. Despite the best interventions achieved by surgery, radiotherapy, and chemotherapy, the median overall survival time is still only 14.6 months [1]. Many innovative strategies, including immunotherapy, are under investigation in the hope of making a breakthrough in glioblastoma treatment. Some immunotherapy studies have demonstrated efficacy in establishing tumor-specific immunity against mouse glioma but there has been only limited success in clinical settings [2]. In spite of these dilemmas, it is certain that there are long-term survivors of glioblastoma. We have hypothesized that, in some cases, adaptive immunity of the host may ameliorate glioblastoma progression. Activating this specific immunity may lead to potential immunotherapy against glioblastoma.

To test this hypothesis, we focused on OX40 and OX40 ligand (L), both of which are known to evoke a key signal for long-lasting immunity [3]. OX40 is a member of the tumor necrosis factor receptor superfamily and interactions between OX40 and OX40L act as costimulatory signals [4,5]. We [4,5] and others have extensively studied the functions of OX40 and OX40L in the immune system, including the generation of memory T cells [3,6,7] and the stimulation of CD4 T cells, CD8 T cells, and natural killer T cells [5,8,9]. Triggering OX40 signaling has been demonstrated in various mouse tumor models [10-14] as a potential antitumor therapy and its translation to clinical trials is under investigation [8,15]. However, the mechanism by which OX40 signaling in glioblastoma regulates the antitumor adaptive immunity of the host remains unknown.

OX40L expression is believed to be restricted to cells related to the immune system and its surroundings [4,16-19]. However, we have discovered that OX40L is expressed in human glioblastoma cells. We therefore addressed the possible role of OX40L expressed in glioblastoma cells and immunotherapy targeting OX40 signaling.

## Methods

### Patients and glioblastoma specimens

Informed consent was obtained from all subjects. The ethics committee of Tohoku University School of Medicine approved the study. Tissue specimens were obtained from patients with primary glioblastoma (n = 110) and grade III glioma (n = 34). Among the 110 patients with glioblastoma, 79 patients underwent gross total resection. Human T cells were obtained from healthy human donors.

### PCR amplification

RNA was extracted from human glioblastoma tissue and from five human glioblastoma cell lines, using the RNeasy Lipid Tissue Mini Kit (Qiagen Science, Germantown, MD). Reverse transcription was performed using the High Capacity RNA-to-cDNA Kit (Applied Biosystems, Carlsbad, CA). The expressions of OX40L mRNA and the internal control β-actin mRNA was analyzed using the TaqMan Gene Expression Assays (tumor necrosis factor ligand, superfamily, member 4, Assay ID: Hs00182411_m1, and β-actin Control Reagents, respectively, Applied Biosystems). A mixture (20 μl) of cDNA, the TaqMan Fast Advanced Master Mix (Applied Biosystems) and each probe was subjected to amplification with StepOnePlus Real-Time PCR Systems (Applied Biosystems), according to the manufacturer’s instructions.

### Immunohistochemistry

Immunostaining was performed by the streptavidin-biotin method using a Histofine kit (Nichirei co Ltd. Tokyo, Japan). Tag34, a mouse monoclonal antibody against human OX40L [20] was used as the primary antibody (1:100). Paraffin-embedded sections of human glioblastoma specimens (n = 23) were deparaffinized with xylene and absolute ethanol. Antigen retrieval was performed by heating the slides with a microwave in 10 mM EDTA (pH8) for 10 minutes. A172 human glioblastoma cells were cultured on Millicell EZ slide (Millipore, Billerica, MA) and fixed by 4% paraformaldehyde at room temperature for 15 minutes. Both staining was followed by previously reported protocol [21]. As for the positive control, we used tissues of human skin psoriasis [21] and for the negative control, normal mouse IgG was used. The degree of OX40L expression was scored by I.S. and two pathologists (Y.S. and H.S.).

### Survival analysis

The Mann–Whitney U test was used for comparison of continuous variables. To analyze whether OX40L mRNA expression was associated with progression-free survival (PFS), we used the univariate Cox proportional hazard model, with relative OX40L expression being the only covariate. OX40L expression data were distributed exponentially and were logarithmically transformed. When OX40L expression was zero, i.e., undetectable, we imputed 0.001 as the minimum value before transformation. The starting point for PFS and overall survival (OS) was the day of surgery. Tumor progression at the last follow-up examination or death was the end point for PFS and OS. Statistical analyses were performed with R version 3.0.2, and *P* values of <0.05 were considered statistically significant.

### Functional analysis of OX40L expressed in human glioblastoma

Five human glioblastoma cell lines, U87, U251, U373, T98 and A172, were used in this study. Ethylendiamine tetraacetic acid (EDTA) solution was used to detach cells without altering the structure of OX40L protein. For detecting OX40L expression, antibodies specific for biotinylated Tag34 were used, followed by PE-streptavidin. Analysis was performed using FACS CantoII cytometer and FACS Diva software (BD Bioscience, Franklin Lakes, NJ). In the series of experiments, analyzing the effect of hypoxia on OX40L expression, A172 cells were cultured for 72 h under hypoxic (1.5% O_2_) or normoxic (21% O_2_) conditions. A172 cells were analyzed for OX40L mRNA and protein expression. A172 cells cultured on chamber slides were used for immunohistochemical analysis of OX40L expression, as described above. Cell culture conditions are described in the Additional file 1.

Human CD4 cells (1 × 10^5^) obtained from healthy human donors were cocultured with irradiated A172 cells (3 × 10^4^) in 100 μl of medium per well and either the Tag34 or IgG antibody (20 μg/ml each), under hypoxia or normoxia, in anti-CD3-antibody-coated 96-well plates (BioLegend, San Diego, CA). Irradiated A172 cells were prepared by irradiating 1 × 10^7^ cells seeded in 1 ml PBS, in a 6-cm dish. Anti-CD3-antibody-coated plates were used to stimulate naïve T cells to express OX40 [5]. After 72 h of incubation under normoxia, the supernatant was used for ELISA to measure interferon (IFN)-γ. Human CD4-positive cells (1 × 10^5^) were pretreated with carboxyfluorescein succinimidyl ester (CFSE) (Molecular Probes, Eugene, OR) and were detected in the fluorescein isothiocyanate fraction. The proliferation of activated CD4 cells was followed with flow cytometry. Details are in the Additional file 1. For cell sorting, MicroBeads and the AutoMACS system (Miltenyi Biotec, Gladbach, Germany) were used to isolate human CD4 cells from healthy human blood.

### Mouse cell lines

The mouse cell lines used were GL261 glioma cell line [22], generously provided by Dr. Masaki Toda, Keio University and NSCL61 glioma-initiating cell-like cell line [23], generously provided by RIKEN (Kobe, Japan). NSCL61 cells were established by introducing an oncogenic *HRas*^*L61*^ in *p53*-deficient neural stem cells and were confirmed to retain features of glioma-initiating cell-like cells [23]. Mouse OX40L cDNA or a parent vector (empty vector) was transduced in GL261 cells, using retroviruses to create GL261 cells expressing OX40L (GL261-mOX40L) or empty vector (GL261-mock). The expression of mouse OX40L was analyzed by the flow-cytometry with PE-conjugated anti-mouse OX40L antibody (eBioscience, San Diego, CA). Cell culture and transfection are described in the Additional file 1.

### Mouse models of glioma

All animal experiments were reviewed and approved by the Animal Care and Use Committee, Tohoku University School of Medicine and performed in accordance with institutional ethical guidelines. Six to eight-week-old, female, C57BL/6 mice and BALB/c nude mice were purchased from SLC Japan, Inc. (Shizuoka, Japan). The generation of OX40-knockout (OX40KO) mice was described previously [24,25]. After anesthesia, each mouse was injected either with GL261 cells (1 × 10^5^ or 2 × 10^5^) or NSCL61 cells (1 × 10^4^) into the right striatum. A small number of NSCL61 cells were inoculated because of their aggressive growth potential. Details are described in the Additional file 1.

Mouse brain frozen sections were stained with either the anti-CD4 or the anti-CD8 antibody (Abcam, Cambridge, MA), followed by anti-rat IgG Zenon® Alexa 568 (Invitrogen, Carlsbad, CA). Details are in the Additional file 1.

### OX40 triggering on T-cell activation

Lymphocytes (1 × 10^5^) harvested from the spleens of mice vaccinated with OX86 and irradiated GL261 cells were cultured with irradiated GL261 cells (3 × 10^4^) for 3 days, to assess GL261 cell-specific T-cell activation. Supernatants were used for ELISA to detect mouse IFN-γ (BD OptEIA; BD Bioscience). MicroBeads and the AutoMACS system (Miltenyi Biotec, Gladbach, Germany) were used to isolate lymphocytes from mouse spleens. For detecting the effector T cells, CD4-Pacific Blue, CD44-APC and CD62L-FITC (eBioscience) were used. FACS analysis was performed, as described above. Treg cells (1 × 10^5^) collected from spleens of untreated wild-type mice were cocultured with either OX86 or IgG antibodies, under hypoxia or normoxia, in anti-CD3 antibody-coated 96-well plates. Supernatants were used to measure IL-10 with ELISA (R&D Systems, Inc.).

### Vaccination therapy in mice bearing transplanted glioma

For vaccination, mice received subcutaneous injections of the mixture that contained tumor cell lysates and 250 μg of purified rat IgG reagent (Sigma–Aldrich) or OX86, a mouse OX40 agonistic antibody, into the left flank. To prepare the tumor cell lysates, cells (1 × 10^7^) were seeded in 1 ml of PBS in a 6-cm dish and irradiated (5,000–7,000 rad). Irradiated GL261 cells (5 × 10^5^) or NSCL61 cells (5 × 10^4^) were used as cell lysates. The OX86 antibody was obtained from the supernatant of OX86 hybridoma (European Collection of Cell Cultures). For comparison, mice were vaccinated with intraperitoneal injection of OX86 or IgG antibody. Vaccination was performed twice at a 5-day interval. In the study of tumor therapy, mice bearing the transplanted GL261 or NSCL61 cells were treated with respective tumor lysates in combination with OX86 or IgG control antibody. Mice were monitored daily for survival and general health. To assess the effect of memory T cells, mice with intracranial GL261 cells, which survived after OX86 vaccination, were reinjected with GL261 cells (2 × 10^5^) into the contralateral hemisphere.

### Statistical analysis

All statistical analyses, excluding human survival analyses, were performed with the Prism software (GraphPad Software, San Diego, CA). All *P* values were two tailed and *P* values of <0.05 were considered statistically significant.

## Results

### Expression of OX40L in human glioblastoma

**Figure 1:**
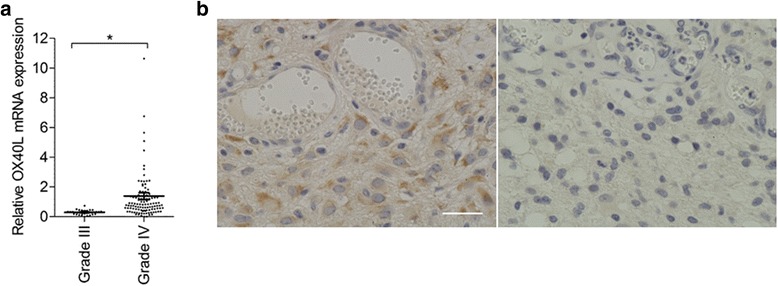
**Expression of OX40L in human glioblastoma. a**. OX40L mRNA expression in grade IV glioma (glioblastoma) (n = 110) and grade III glioma (n = 34, *P < 0.05). **b**. Representative immunohistochemical images of glioblastoma specimens stained with Tag34, anti-human-OX40L antibody (brown). The left panel shows a tumor tissue section with many OX40L-positive cells and the right panel shows a tumor tissue section with limited positive cells. Scale bar, 40 μm.

To explore the role of OX40 signaling in antitumor adaptive immunity of human glioblastoma, we initially analyzed whether OX40L mRNA was expressed in excised tumor specimens. The quantitative PCR analysis showed that OX40L mRNA expression levels were significantly higher in glioblastoma (grade IV glioma) than in grade III glioma (Figure 1a). However, no significant difference was detected in OX40L mRNA expression levels between the 79 patients with gross total resection and the 31 patients without gross total resection (*P* = 0.29, data not shown). Thus, OX40L expression and the extent of surgical removal did not correlate with each other.

The immunohistochemical analysis showed that OX40L protein was expressed in cells with atypical nuclei (Figure 1b, left), indicating that OX40L protein was expressed in glioblastoma cells. However, there were cases in which OX40L protein expression was undetectable (right panel). In fact, among the 23 glioblastoma specimens analyzed, only six samples were strongly positive for OX40L expression (26%).

Because OX40L is a potent immunoinducer, we next explored its effect on the prognosis of glioblastoma patients. The univariate Cox proportional hazard model showed that higher OX40L expression levels were associated with longer PFS in patients with glioblastoma (n = 110), although the difference was not statistically significant (Hazard Ratio per log-relative OX40L expression, 0.898; 95% confidence interval, 0.775–1.041; *P* = 0.155). Importantly, among the glioblastoma patients who underwent gross total resection (n = 79), higher OX40L expression was associated with longer PFS (Hazard Ratio per log-relative OX40L expression, 0.776; 95% confidence interval, 0.611–0.985; *P* = 0.037). In contrast, OX40L expression was not associated with PFS in the 31 patients with glioblastoma, who did not undergo gross total resection. Moreover, there was no statistical significance between OX40L expression level and overall survival (OS), irrespective of the gross total resection (among gross totally resected patients, hazard ratio per log-relative OX40L expression, 0.936; 95% confidence interval, 0.762–1.15; *P* = 0.531). These results suggest that gross total resection may resolve a certain immunosuppressive condition in glioblastoma to enhance the growth inhibitory effect of OX40 signaling.

### Functional implication of OX40L expressed in glioblastoma

**Figure 2:**
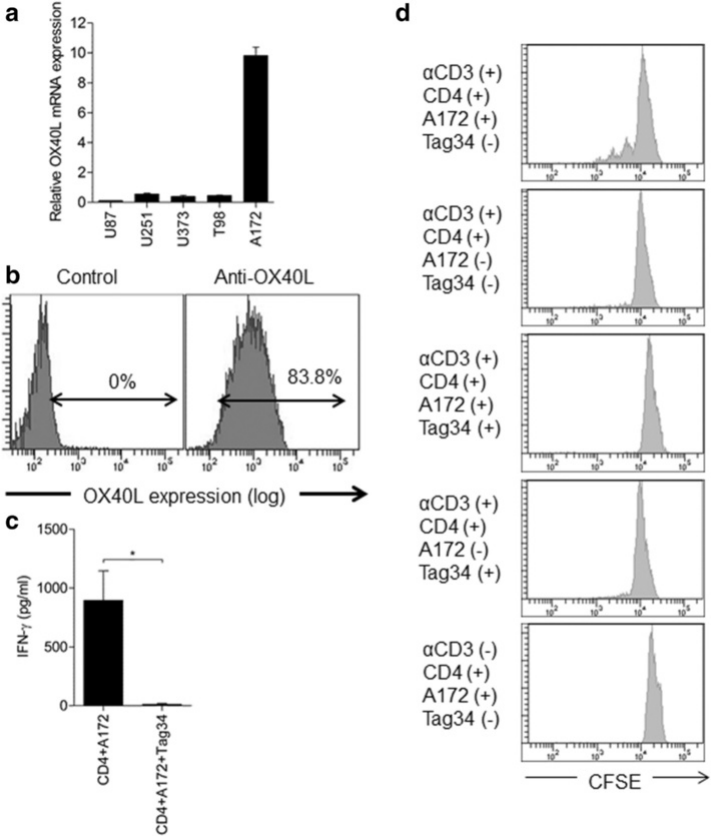
**Endogenous OX40L in human glioblastoma and its functional implication. a**. Quantitative PCR of OX40L mRNA expression in five glioblastoma cell lines. **b**. OX40L protein expression in A172 cells, as determined by flow cytometry. **c**. Production of IFN-γ from human CD4-positive cells, cocultured with irradiated A172 cells. Tag34, a blocking antibody against human OX40L (*P < 0.05), under anti-CD3 coated plates. **d**. CFSE staining of human CD4-positive cells detected by flow cytometry. The top panel shows the cell division pattern of CD4-positive cells that were cocultured with irradiated A172 cells in anti-CD3 antibody-coated plates. Compared with the top panel, either the absence of A172 (2nd panel) or the addition of Tag34 (3rd panel), Tag34 only (4^th^ panel) or the absence of anti-CD3 (5^th^ panel), resulted in the loss of the cell division patterns.

To explore the functional consequence of OX40L expression in human glioblastoma, we searched for human glioblastoma cell lines that express OX40L. Out of five human glioblastoma cell lines, OX40L mRNA was highly expressed in A172 cells (Figure 2a). We then confirmed OX40L protein expression in A172 cells by flow cytometry (Figure 2b) but were unable to detect OX40 expression (data not shown). Accordingly, irradiated A172 cells, expressing OX40L, were used as a stimulant of OX40 and cocultured in the anti-CD3-coated plate with human CD4 cells, isolated from healthy adult volunteers. The condition medium from this coculture contained large amounts of IFN-γ (Figure 2c) but this IFN-γ production was blocked with Tag34, a blocking antibody to human OX40L. These results indicate CD4 cell activation by endogenous OX40L. In CFSE staining, we showed that the proliferation of human CD4 cells depended on the presence of both A172 cells and anti-CD3 antibody (Figure 2d). These results suggest that OX40L, expressed in glioblastoma cells, may induce T-cell activation, thereby enhancing antitumor adaptive immunity.

### OX40 signaling restricts glioma progression in mouse models

**Figure 3:**
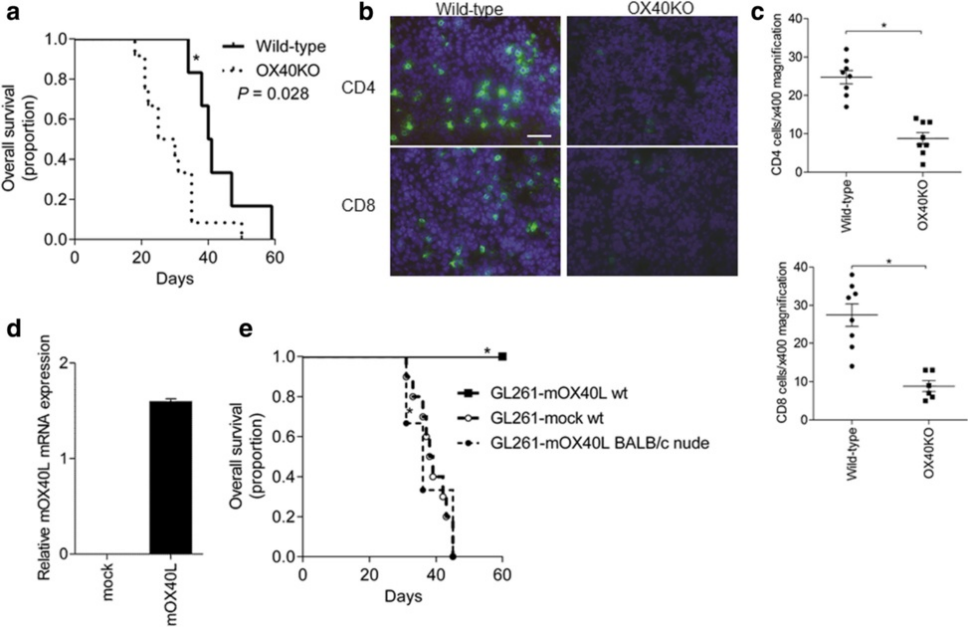
**OX40 signaling in mouse models of glioma. a**. Survival curves of OX40KO mice (n = 12) and wild-type mice (n = 6) with intracranial GL261 cells. OX40KO mice bearing the GL261 cell transplant were associated with significantly shorter survival than that of wild-type mice (*P* = 0.028). **b**. CD4 and CD8 cells (green) at the sites of the GL261 cell transplants of wild-type and OX40KO mice. Scale bar, 40 μm. **c**. Numbers of CD4 and CD8 cells, counted under 400× magnification (mean ± SEM) (*P < 0.05). **d**. Transfection of G261 cells with retrovirus harboring OX40L cDNA or empty vector. The expression of mouse OX40L (mOX40L) was confirmed by quantitative PCR. **e**. Survival curves. GL261-mOX40L cells were injected into the brain of wild-type mice (wt, n = 10) or BALB/c nude mice (n = 3). GL261-mock cells were injected into the wild-type mouse brain (mock wt, n = 10) for control (*P < 0.0001).

To explore the protective role of OX40 signaling in glioma progression, we established a mouse model of glioma. GL261 mouse glioma cells, lacking OX40L (Additional file 2: Figure S1a), were injected into the brains of wild-type and OX40KO mice. The survival time was significantly longer in the wild-type mice than in the OX40KO mice (*P* = 0.028), suggesting that the presence of OX40 signaling may restrict glioma progression (Figure 3a). To explore the underlying mechanism, we then analyzed CD4 and CD8 cell infiltration in the intracranial tumor of wild-type mice and OX40KO mice, 21 days after GL261 cell implantation. The infiltration of CD4 and CD8 cells was apparent within the tumors of wild-type mice (Figure 3b), and the number of infiltrating CD4 and CD8 cells was significantly higher than that in OX40KO mice (Figure 3c). These results indicate that the absence of OX40 signaling can generate only a weak adaptive immunity, resulting in the impairment of immune cells in the transplanted tumor, which may lead to continuous tumor growth in OX40KO mice. Taken all together, OX40 signaling is important in antitumor immunity.

To confirm the role of OX40 signaling in mouse glioma, we next used GL261-mOX40L cells that were forced to express exogenous OX40L. The GL261-mOX40L cells or GL261-mock cells (Figure 3d) were transplanted into the wild-type mouse brain to establish the model that simulates human glioblastoma expressing OX40L. The mice transplanted with GL261-mOX40L cells did not die for at least 60 days after the cell transplantation (Figure 3e), whereas all mice transplanted with GL261-mock cells died by 50 days. GL261-mOX40L cells were also injected subcutaneously into the wild-type mice but none of them formed a tumor (data not shown). These results suggest that OX40L expression may restrict the growth of transplanted cells, thereby conferring survival advantage on the host. Moreover, the BALB/c nude mice bearing intracranial GL261-mOX40L cells died within 50 days (Figure 3e). The overall survival was indistinguishable between the nude mice bearing GL261-mOX40L cells and the wild-type mice bearing GL261-mock cells. Thus, GL261-mOX40L cells were effectively eliminated probably by OX40L-induced immunoreactive cells before forming a tumor mass.

### OX40 triggering induces T-cell activation

**Figure 4:**
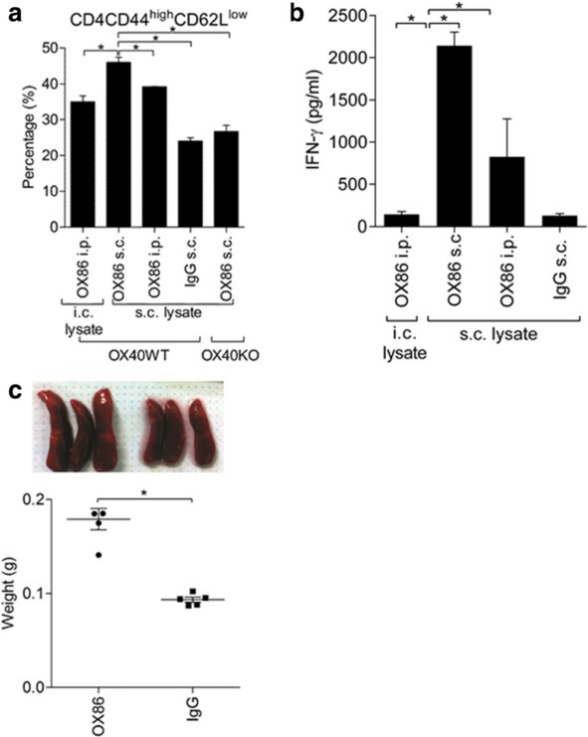
**Subcutaneous OX86 vaccination in mice. a**. Various vaccinations to induce effector T cells, determined by flow cytometry (mean ± SEM, *P < 0.05). **b**. IFN-γ production determined by ELISA (mean ± SEM, *P < 0.05). Lymphocytes from vaccinated mice were stimulated with irradiated GL261 cells, and the secreted IFN-γ was measured in the conditioned medium. **c**. Images and weights of spleens isolated from mice 3 days after vaccination with OX86 s.c. or IgG s.c., administered twice with a 5-day interval (n = 5) (mean ± SEM, *P < 0.05).

We have shown a positive correlation between OX40 signaling and antitumor adaptive immunity and thus hypothesized that OX40 triggering may have therapeutic potential against glioma. Accordingly, OX86, a mouse OX40 agonist antibody, was used to enhance OX40 signaling in mice. We first evaluated several vaccination procedures to determine the ideal strategy for immunostimulation by identifying effector T cells (CD4, CD44^high^, CD62L^low^) and measuring the production of IFN-γ using lymphocytes obtained from the spleen, 7 days after vaccination. The tested vaccination procedures included the following: 1. intracranial injection (i.c.) of irradiated GL261 cells as tumor lysates, with intraperitoneal OX86 injection (OX86 i.p.); 2. subcutaneous injection (s.c.) of both irradiated glioma cells and OX86 (s.c. lysate and OX86 s.c.); 3. s.c. lysate with OX86 i.p.; 4. s.c. lysate and s.c. IgG. Among the four vaccination methods, the highest induction of effector T cells was achieved in the mice stimulated with s.c. lysate and s.c. OX86 (Figure 4a and Additional file 3: Figure S2). Importantly, the subcutaneous OX86 vaccination did not increase the population of effector T cells in OX40KO mice, indicating that OX40 signaling is required for the observed immunostimulation (Figure 4a). In addition, the same lymphocytes were restimulated with irradiated GL261 and the produced IFN-γ was measured (Figure 4b). T cells stimulated by subcutaneous OX86 vaccination exhibited the highest IFN-γ production. For the negative control, we confirmed that IFN-γ production was undetectable in irradiated GL261 cells alone or T cells without irradiated GL261 cells (data not shown). Thus, the subcutaneous OX86 vaccination efficiently induced immunostimulation, the degree of which was higher than that of the intraperitoneal injection (systemic administration), generally used in most of the earlier reports. In fact, the size of the spleen was larger and heavier after the subcutaneous OX86 vaccination than after the subcutaneous IgG vaccination (Figure 4c), confirming the efficient immunostimulatory effect of OX86. Accordingly, we used the subcutaneous OX86 vaccination with irradiated tumor cells in the subsequent experiments.

### OX40 triggering as immunotherapy in mouse glioma models

**Figure 5:**
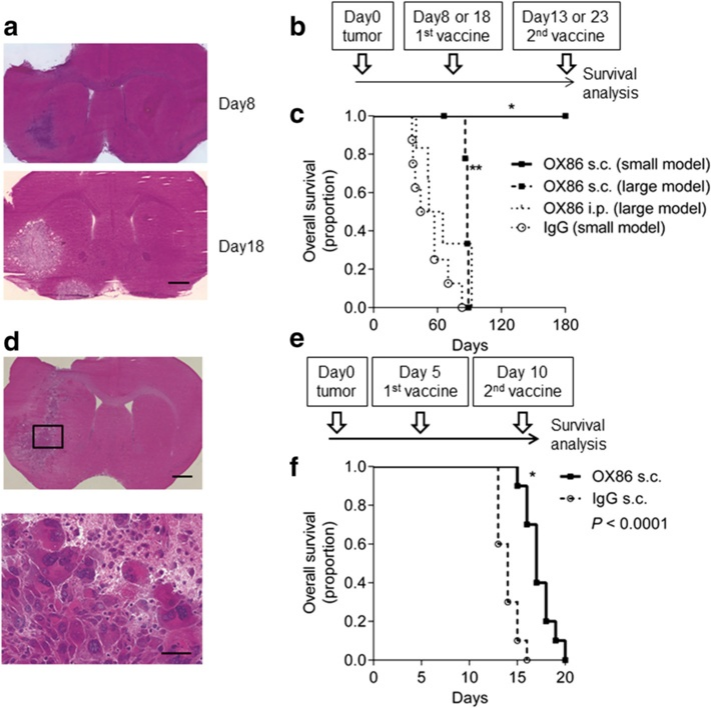
**OX40 triggering as therapeutic vaccination therapy. a**. Representative hematoxylin and eosin (H&E) staining of intracranial tumors with GL261 cells. Day 8 (small model) and Day 18 (large model). Scale bar, 1 mm. **b**. Treatment schedules for small and large model mice. **c**. Survival curves. OX86 s.c. small model mice (n = 6) vs. IgG s.c. (n = 8) small model mice (*P = 0.0004) or OX86 s.c. large model mice (n = 9) (*P* = 0.0009). OX86 s.c. large model mice vs. IgG s.c. (*P < 0.0001). s.c. lysate with OX86 i.p. (n = 6) did not prolong the survival of mice in large model. **d**. H&E staining of intracranial tumors with NSCL61 (Upper scale bar, 1 mm; lower, 400× magnification of boxed area, scale bar, 40 μm). **e**. Treatment schedules for NSCL61 models. **f**. Survival curves of NSCL61 models. OX86 s.c. model mice were associated with prolonged survival than IgG s.c. model mice (n = 10 each, *P < 0.0001).

To investigate the antitumor efficacy of the subcutaneous OX86 vaccination, we conducted a survival analysis of wild-type mice bearing transplanted GL261 cells. The mixture of OX86 or IgG and irradiated GL261 cells was subcutaneously injected to treat mice with smaller glioma (small model), established eight days after intracranial GL261-cell inoculation (Figure 5a), twice at an interval of 5 days (Figure 5b). All OX86-vaccinated mice survived during the observed period of 180 days, whereas all IgG-vaccinated mice died by 70 days (vs. IgG; *P* = 0.0004, Figure 5c). Furthermore, subcutaneous vaccination of OX86 with irradiated tumor cells was effective even in mice with larger glioma (large model), established 18 days after GL261-cell inoculation (vs. IgG; *P* < 0.0001, Figure 5c). Mice with smaller glioma, which survived for 180 days, received a rechallenge of GL261 cells into the contralateral brain to examine memory immunity. None of these rechallenged mice died, indicating established, long-lasting memory immunity by OX40 triggering. In contrast, mice with larger glioma, which were treated with s.c. lysate with OX86 i.p., showed better survival than the subcutaneous IgG-vaccinated group, however, the difference was not significant (vs. IgG; *P* = 0.21; Figure 5c). These observations indicated that targeting OX40 signaling by subcutaneous vaccination is a potential antitumor therapy.

To further confirm this therapeutic effect, we conducted survival analysis in another glioma model. Glioma-initiating cell-like cells have been considered to regulate tumor initiation and formation [26] and are known to be resistant to treatment [27]. Therefore, we assessed the antitumor effect of the subcutaneous OX86 vaccination in mice with the intracranial transplantation of NSCL61 cells (Figure 5d and e) [23]. NSCL61 cells do not express OX40L, as judged by flow cytometry (Additional file 2: Figure S1b). Hematoxylin and eosin staining of intracranial NSCL61 cells showed aggressive features with many atypical cells (Figure 5d). In fact, all mice bearing intracranial NSCL61 cells died by 20 days after transplantation, despite the subcutaneous OX86 vaccination (Figure 5e and f); namely, their survival time was much shorter than that of the mice bearing intracranial GL261 cells (see Figure 5c). These results suggest that NSCL61 cells were more aggressive in proliferation and resistant to the subcutaneous OX86 vaccination than GL261 cells. However, the subcutaneous OX86 vaccination conferred a significant survival advantage on the mice bearing intracranial NSCL61 cells, compared with control IgG vaccination (*P* < 0.0001, Figure 5f). Thus, the subcutaneous OX86 vaccination had a strong antitumor effect in two mice glioma models.

**Figure 6:**
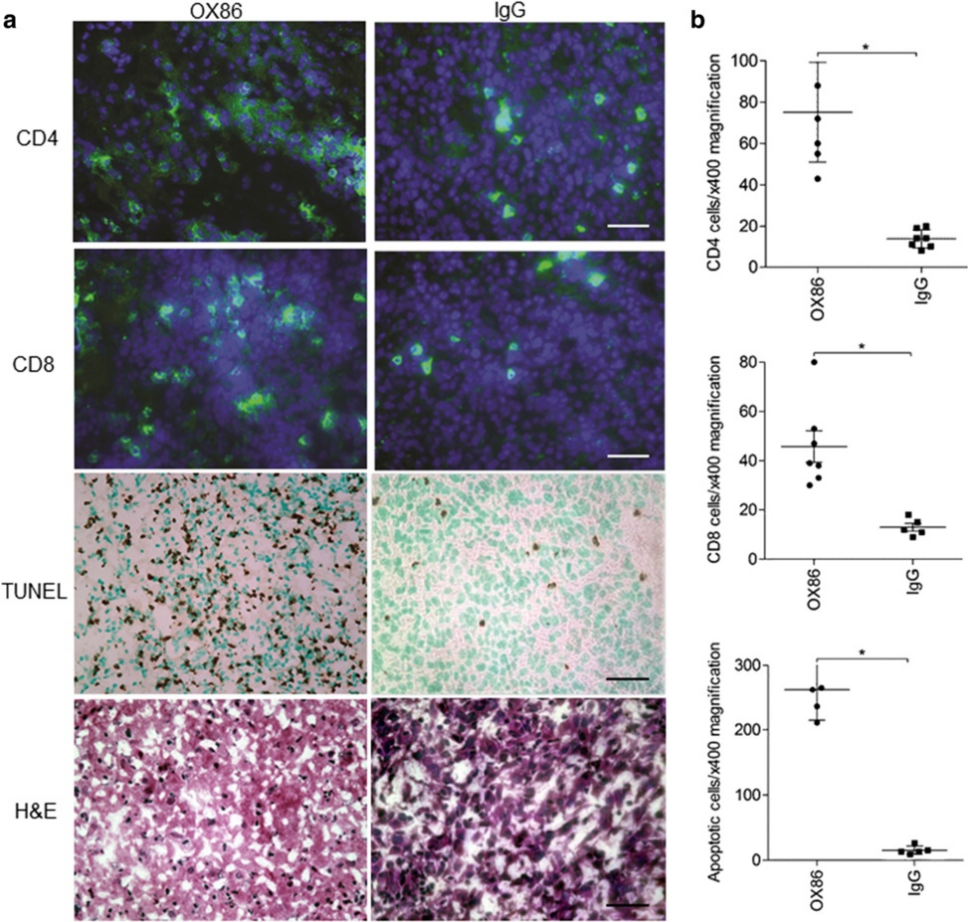
**Accumulation of CD4 and CD8 cells at the transplanted glioma cells after OX86 vaccination. a**. Representative image of intracranial GL261 cells in mice received subcutaneous OX86 or IgG vaccination. CD4 and CD8 cells (green), DAPI (blue), apoptotic cells (brown) by TUNEL, and H&E. Scale bar, 40 μm. **b**. Numbers of CD4 cells, CD8 cells, and apoptotic tumor cells (under 400× magnification, mean ± SEM, *P < 0.05).

To investigate the cellular basis for the antitumor effect of subcutaneous OX86 vaccination, we analyzed the presence of CD4 and CD8 cells within the transplanted GL261 cells. Brain tissue sections, prepared from the large-model mice that received the second vaccination, were examined by immunohistochemistry for CD4 and CD8 cells. The subcutaneous OX86 vaccination caused a significant increase in the numbers of CD4 cells, CD8 cells, and apoptotic cells, compared with the subcutaneous IgG vaccination (Figure 6a and b). Notably, the increase in CD4 and CD8 cell numbers was higher than that in the transplanted GL261 cells in wild-type mice without the vaccination (see Figure 3b and c). Large areas of necrotic brain tissues were also found in mice treated with the subcutaneous OX86 vaccination. Thus, the subcutaneous OX86 vaccination recruited a large number of immune cells to the tumor microenvironment, thereby enhancing the antitumor adaptive immunity and glioma cell apoptosis.

### Paradoxical effects of OX40L on glioblastoma growth

**Figure 7:**
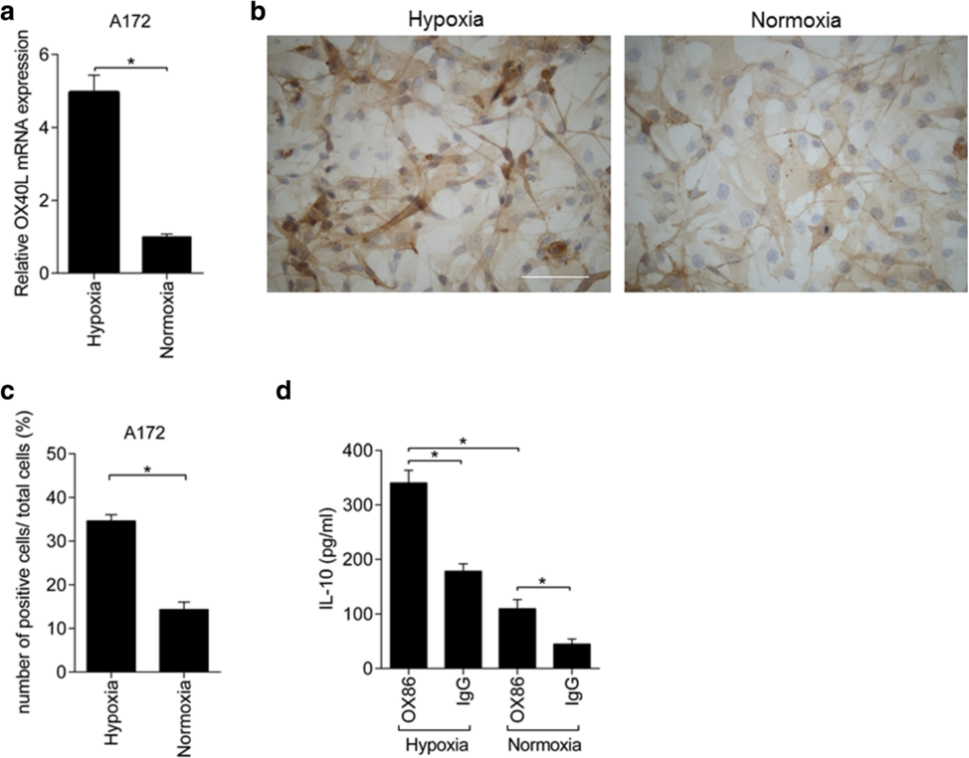
**Pro-tumor immunity of OX40 signaling. a**. The expression of OX40L mRNA was induced in A172 glioblastoma cells under hypoxia (*P < 0.05). **b**. Representative immunohistochemistry of OX40L protein in A172 cells. Under hypoxia (left panel), under normoxia (right panel). Scale bar, 40 μm. **c**. Numbers of OX40L positive cells/ total cells, counted under 200× magnification (mean ± SEM, *P < 0.05). The data are shown as % positive cells per total cells. **d**. Production of IL-10 protein from mouse Treg cells, as determined by ELISA (n = 5) (mean ± SEM, *P < 0.05).

Human glioblastoma cells express OX40L, despite the fact that OX40L functions as a strong antitumor immunoinducer, providing a survival benefit to the host. The immunostimulatory potential of OX40L raises the basic question, as to why human glioblastoma expresses OX40L. Some pro-tumor effects, i.e., benefits for the glioblastoma cells, must exist for glioblastoma to gain OX40L expression. We hypothesized the existence of an on–off switch, which changes the balance between the antitumor and the pro-tumor effects of OX40L. One candidate of such a switch is hypoxia, because hypoxia is a condition found in the microenvironment of glioma [28] and the survival benefit of OX40L expression was detected only in patients who underwent gross total resection of glioblastoma. Taking the advantage that OX40L is expressed in A172 human glioblastoma cells (see Figure 2), we analyzed the effect of hypoxia on OX40L expression. PCR analysis demonstrated that hypoxia induced OX40L mRNA expression in A172 cells (Figure 7a). OX40L protein expression was then analyzed with immunohistochemistry (Figure 7b). Immunopositivity was identified in cytoplasm and occasionally in cell membrane. The percentage of OX40L-positive cells was significantly higher in A172 cells cultured under hypoxia than in cells cultured under normoxia (Figure 7c). In addition, cytoplasmic immunostaining was apparently higher under hypoxia than under normoxia. Thus, hypoxia induces OX40L expression in glioblastoma cells, which is consistent in part with our hypothesis that the hypoxic microenvironment may be involved in OX40L expression in glioblastoma.

Consequently, we focused on Treg cells that express OX40 [18] and are also abundant in the glioblastoma microenvironment [18,29,30]. Mouse Treg cells were cultured with OX86 or the control IgG antibody, under hypoxia or normoxia, in anti-CD3-antibody-coated plates. The level of IL-10, an immunosuppressive cytokine, was significantly increased in the conditioned medium of Treg cells with OX86 treatment under hypoxia, compared with that under hypoxia with IgG treatment or under normoxia with OX86 treatment (Figure 7d). Thus, hypoxia significantly increased IL-10 production from Treg cells, the degree of which was further enhanced by OX86. Taken together, these results suggest that glioblastoma may cooperate with Treg cells via OX40L, thereby enhancing IL-10 production. IL-10 in turn generates the immunosuppressive microenvironment. Such pro-tumor effects caused by Treg cells may be enhanced under hypoxic conditions.

## Discussion

Here, we show for the first time that glioblastoma cells express OX40L, which activates OX40 signaling and strengthens antitumor adaptive immunity. Furthermore, we show that higher OX40L expression is significantly associated with prolonged progression-free survival. An *in vivo* study, using OX40KO mice and GL261-mOX40L cells, showed the importance of OX40 signaling in antitumor immunity. Collectively, adaptive immunity evoked by OX40 signaling has the potential to suppress the proliferation of glioblastoma cells. OX40 triggering has been reported as an effective anti-cancer therapy, in which systemic i.p. administration was mainly used [12,13,31]. We found that s.c. injection of OX86 and tumor lysate induced stronger antitumor immunity than systemic i.p. injection, thereby providing persistent long-term antitumor effects. In terms of T-cell activation, subcutaneous OX86 vaccination, a mixture of both OX86 and tumor antigen (irradiated GL261 cells), may be an efficient method for generating tumor-specific T-cell activation.

The expression of OX40L in glioblastoma suggests a hitherto unrecognized role of OX40 signaling. It was initially surprising to us that glioblastoma expresses endogenous OX40L, which may result in self-attacking. To resolve this paradox, we focused on oxygen tension and thus discovered the potential pro-tumor effects of OX40 signaling. Under hypoxia, which simulates the tumor microenvironment, OX40 triggering induced IL-10 production from Treg cells, which may provide a suitable environment for tumor progression. Treg cells consistently express OX40 [18] and are known to accumulate in the microenvironment of glioblastoma [29,30]. Thus, expressing OX40L might be a strategy of glioblastoma to create the immunosuppressive environment with the help of Treg cells. In fact, OX40L expression was upregulated in A172 cells under hypoxia. Our data also showed that OX40L-expressing glioma cells could not form a tumor mass in wild-type mouse brain. Kaneko et al. presented anti-lymphoma immunity by introducing OX40L into lymphoma cells, which was consistent with our data [32]. Glioblastoma cells probably express OX40L only after tumor bulk reaches a critical size, which could generate the hypoxic environment suitable for tumor progression. In this context, we observed the survival benefit of OX40L only in patients that were able to undergo gross total removal. Thus, this immunostimulative aspect may be achieved via resolving the hypoxic microenvironment of glioblastoma.

Around 26% of the analyzed glioblastoma tissue presented high OX40L, indicating this phenomenon is not applicable to all glioblastoma. We showed that OX40 signaling achieved opposite functions, immunoinduction and immunosuppression, depending on oxygen tension. In fact, Ruby et al. reported that OX40 triggering had two opposite effects on Treg cells, depending on the local cytokine milieu [33]. Moreover, ovarian cancer produced CC-chemokine ligand 28 under hypoxic conditions, resulting in Treg cell recruitment and the induction of tumor angiogenesis through VEGFA secretion [34]. Thus, difference in the oxygen tension may account for the dual functions of OX40L.

There are a few limitations in the present study. First, for the mouse survival analysis, depletion analysis was not conducted, although we showed the effective induction of tumor specific antitumor immunity by numerous apoptotic cells. Second, concerning T-cell activation, pathway analysis was not performed. Studies on the downstream pathway of OX40 signals, such as NF-κB [4], need to be conducted. Lastly, T cells used in this study were mainly obtained from the mouse spleen because of the difficulty in the isolation of T cells from glioblastoma tissues or the cervical lymph node. Considering this limitation, we have presented the OX40-mediated immunoinduction using human T cells.

## Conclusion

Here, we demonstrated OX40L expression in human glioblastoma cells. We also showed that the activation of OX40/OX40L signaling, by subcutaneous vaccination, induces strong and long-lasting immunity, with therapeutic antitumor effects using mouse glioma models. This result is in agreement with the clinical survival data on glioblastoma patients. On the other hand, we also provide evidence of crosstalk between glioblastoma cells and Treg cells under hypoxic conditions, which provides glioblastoma cells with immunosuppressive benefits. Thus, immunostimulation and immunosuppression are closely connected within glioblastoma, and targeting of these two aspects may unravel a clue for therapeutic approach against glioblastoma.

## Additional files

Additional file 1: **Supplementary materials are available online.**

Additional file 2: Figure S1. Lack of OX40L expression in mouse glioma cell lines a and b: The expression of OX40L was analyzed by flow cytometry in GL261 glioma cells (a) and NSCL61 glioma-initiating cell-like cells (b). OX40L is not expressed in these cell lines.

Additional file 3: Figure S2. Representative FACS analysis of mouse T cells: T cells were isolated from the spleen of wild-type mice, 7 days after subcutaneous OX86 (OX86 s.c.) or IgG (IgG s.c.) vaccination with GL261 tumor lysates, each administered twice, with a 5-day interval. They were stained with CD4-Pacific Blue, CD44-APC and CD62L-FITC. The proportion of effector T cells was higher in mice with subcutaneous OX86 vaccination than those with subcutaneous IgG vaccination.
