# Supplementary material for: OX40 ligand expressed in glioblastoma modulates adaptive immunity depending on the microenvironment: a clue for successful immunotherapy
Source: Mol Cancer. 2015 Feb 15;14:41. doi: 10.1186/s12943-015-0307-3 (PMC4339477; doi:10.1186/s12943-015-0307-3)
Supplement: Supplementary file 1 — Supplementary materials are available online. [file 12943_2015_307_MOESM1_ESM.docx]

**Supplementary Materials and Methods**

**Cell lines and cell culture**

Human glioblastoma cell lines; U87, U251, U373, T98, and A172, were obtained from DS PHARMA Biomedical (Osaka, Japan), the Health Science Research Resources Bank (Osaka, Japan), ATCC, DS PHARMA Biomedical and RIKEN BRC (Tsukuba, Ibaraki, Japan), respectively. The mouse glioma cell line GL261 [22], generously provided by Dr. Masaki Toda, Keio University, and the mouse glioma-initiating cell-like cell line NSCL61 [23], generously provided by RIKEN (Kobe, Japan), were also used in this study. Cells were cultured in DMEM/F12 supplemented with 10% fetal bovine serum, 1% penicillin, and streptomycin (Gibco, Grand Island, NY). The NSCL61 cells only were maintained in DMEM/F12 supplemented with B27 (Gibco), basic fibroblast growth factor (10 ng/ml) (Peprotech, Rocky Hill, NJ), and epidermal growth factor (10 ng/ml) (Peprotech), as described previously.

Lymphocytes obtained from mouse spleen or human blood were suspended in RPMI 1640 medium (Wako, Osaka, Japan), supplemented with 10% FBS, 1% penicillin, and streptomycin, and were plated in 96-well plates at a density of 1 × 10^5^ cells per well in 100 μl of medium. Ethylendiamine tetraacetic acid (EDTA) solution was used to detach cells without altering the structure of the OX40L protein.

Mouse OX40L or empty cDNA was transduced on GL261 using retroviruses to create GL261 expressing mouse OX40L (GL261-mOX40L) or mock (GL261-mock). To generate cells expressing OX40L, mouse OX40L cDNA [6, 11] was cloned into pQCXIN vector. pQCXIN-mOX40L and pVSV-g were introduced into 293gp cells using CalPhos Mammalian Transfection Kit (Takara bio inc., Shiga, Japan). The conditioned medium containing retroviruses was incubated with GL261 cells that do not express OX40L, supplemented with hexadimethrine bromide (Sigma-Aldrich, Milwaukee, WI). At the last step, positive clones were selected with Geneticin selective antibiotic (G418 sulfate) (Life technologies corporation, Carlsbad, CA) and obtained GL261-OX40L, cultured in DMEM/F12 supplemented with 10% fetal bovine serum, 1% penicillin, and streptomycin (Gibco).

**PCR Amplification, Flow Cytometry, CFSE staining and Sorting**

RNA was extracted from human glioblastoma tissues and from five human glioblastoma cell lines using the RNeasy Lipid Tissue Mini Kit (Qiagen Science, Germantown, MD). Reverse transcription was performed using the High Capacity RNA-to-cDNA Kit (Applied Biosystems, Carlsbad, CA). Expression of OX40L mRNA and the internal control β-actin was analyzed using the TaqMan Gene Expression Assays (Applied Biosystems) using probes from the TaqMan Gene Expression Assays library (tumor necrosis factor [ligand] superfamily, member 4, Assay ID: Hs00182411_m1, and β-actin Control Reagents, respectively, Applied Biosystems). A mixture (20 µl) of cDNA, the TaqMan Fast Advanced Master Mix (Applied Biosystems), and each probe was subjected to amplification with StepOnePlus Real-Time PCR Systems (Applied Biosystems) according to the manufacturer’s instructions.

For detecting the OX40L expression, cultured cells were stained for 20 minutes on ice with biotinylated Tag34 (a mouse monoclonal antibody for human OX40L) [[20]](#_ENREF_23) followed by PE-streptavidin for A172 human glioblastoma cells or with PE-conjugated anti-mouse OX40 ligand (eBioscience, San Diego, CA) for mouse cells (GL261 or NSCL61). Stained cells were subjected to flow cytometric analysis with FACS CantoII cytometer (BD Bioscience) and the proportion was calculated using FACS Diva software (BD Bioscience).

For detecting effector T cells, CD4-Pacific Blue, CD44-APC, and CD62L-FITC (eBioscience) were used and analyzed.

For isolating effector T cells, lymphocytes obtained from wild-type mice vaccinated twice with either OX86 or IgG in various methods were stained with fluorochrome-conjugated antibodies specific for CD4-Pacific Blue, CD44-APC, and CD62L-FITC (eBioscience) and analyzed, as described above. Human CD4-positive cells (1×10^5^) from healthy human donors were pre-treated with carboxyfluorescein succinimidyl ester (CFSE) (Molecular Probes, Eugene, OR) for 5 minutes at 37°C. CFSE labelling is distributed equally between the two daughter cells, when parent cells divide. The CFSE-labeled CD4-positive cells were then co-cultured in anti-CD3 antibody-coated plates with or without irradiated A172 cells (3×10^4^) in the absence or presence of Tag34 (20 μg/ml). The cells were also co-cultured in non-coated plates with A172 cells in the absence of Tag34. After 5 days of co-culture in 100 μl of medium, cells were collected for FACS analyses. CFSE-stained cells were detected in the fluorescein isothiocyanate fraction, and the proliferation of activated CD4 cells was followed with flow cytometry.

For cell sorting, lymphocytes obtained from healthy human blood or mouse spleens were used. Human CD4 cells were selected by Ficoll density gradient separation, followed by anti-CD4 MicroBeads and the AutoMACS system (Miltenyi Biotec, Gladbach, Germany). For the purification of mouse Treg cells, lymphocytes were incubated with biotin-conjugated anti-CD25 antibody (BD PharMingen, San Diego, CA), followed by anti-biotin MicroBeads and separation using the AutoMACS system [18].

**Immunohistochemistry and immunofluorescence**

Mouse frozen brain sections with a thickness of 7-μm were cut through the regions containing the glioblastoma and fixed in acetone for immunohistochemistry and immunofluorescence. Hematoxylin and eosin (H&E) staining was used to assess the tumor burden. After blocking with normal goat serum, sections were incubated with either the anti-CD4 or the anti-CD8 antibody (Abcam, Cambridge, MA) for 2 hours at room temperature. After washing, the sections were incubated with anti-rat IgG Zenon^®^ Alexa 568 (Invitrogen, Carlsbad, CA) for 30 minutes. Nuclei were stained with the VECTASHIELD Mounting Medium with 4’,6-diamidino-2-phenylindole (Vector Laboratories, Inc., Burlingame, CA). Apoptotic cells were detected with the method of terminal deoxynucleotidyl transferase dUTP nick end labeling (TUNEL) (Apoptosis Detection Kit by TUNEL method; WAKO, Osaka, Japan). Finally, 3,3'-diaminobenzidine staining and methyl-green nuclear counterstaining were performed (Vector Laboratories, Inc.). All sections and images were reviewed and scored under a microscope (BZ-9000, Keyence, Inc., Osaka, Japan).

**Mouse Experiments**

After anesthesia with ketamine and xylazine, each mouse was placed in a stereotactic apparatus and a hole was drilled in the skull (0.5 mm forward and 2.5 mm lateral from the bregma), and either GL261 (1×10^5^ or 2×10^5^ cells) or NSCL61 (1×10^4^ cells) cells were injected into the right striatum at the depth of 3.5 mm from the dura using a 10-µl Hamilton syringe with a 26-gauge needle.
