# Supplementary figures and images for: OX40 ligand expressed in glioblastoma modulates adaptive immunity depending on the microenvironment: a clue for successful immunotherapy
Source: Mol Cancer. 2015 Feb 15;14:41. doi: 10.1186/s12943-015-0307-3 (PMC4339477; doi:10.1186/s12943-015-0307-3)

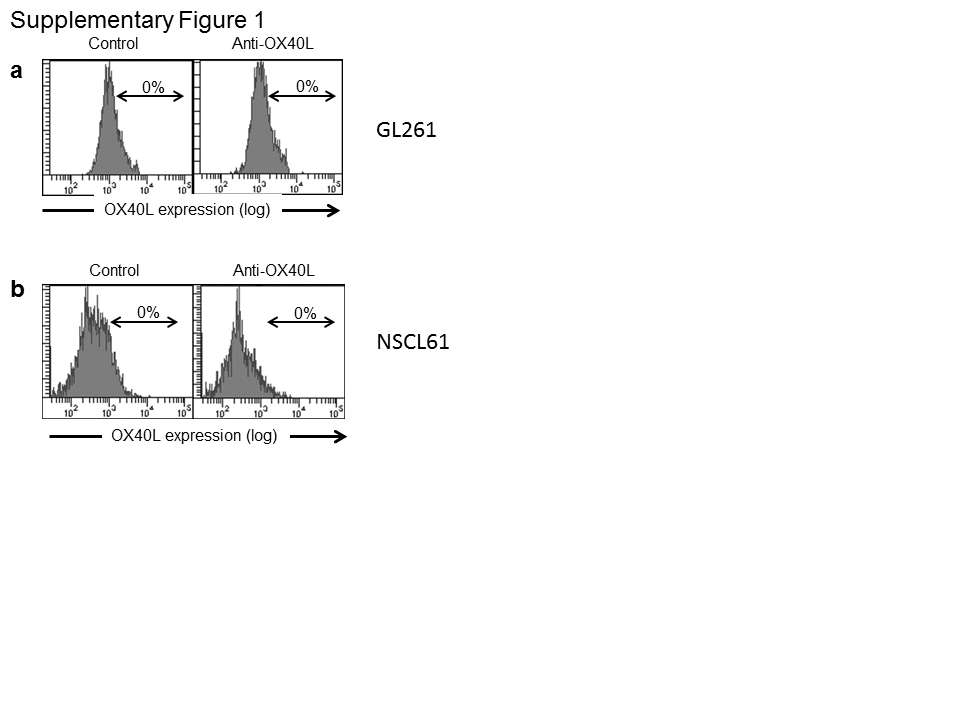

Supplement: Supplementary file 2 — Lack of OX40L expression in mouse glioma cell lines a and b: The expression of OX40L was analyzed by flow cytometry in GL261 glioma cells (a) and NSCL61 glioma-initiating cell-like cells (b). OX40L is not expressed in these cell lines. [file 12943_2015_307_MOESM2_ESM.tiff]

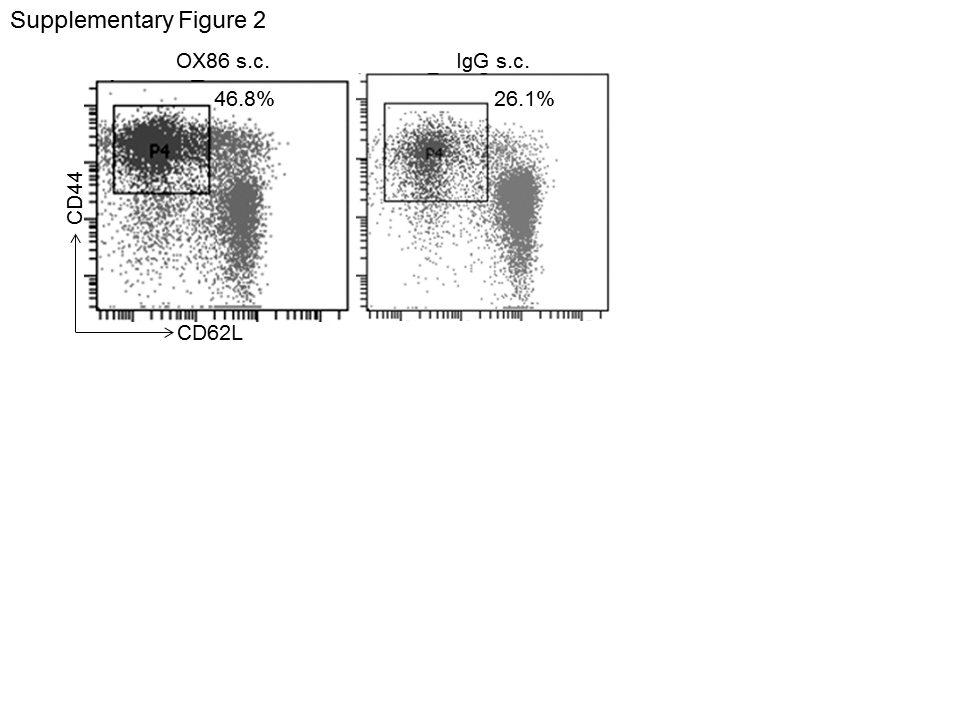

Supplement: Supplementary file 3 — Representative FACS analysis of mouse T cells: T cells were isolated from the spleen of wild-type mice, 7 days after subcutaneous OX86 (OX86 s.c.) or IgG (IgG s.c.) vaccination with GL261 tumor lysates, each administered twice, with a 5-day interval. They were stained with CD4-Pacific Blue, CD44-APC and CD62L-FITC. The proportion of effector T cells was higher in mice with subcutaneous OX86 vaccination than those with subcutaneous IgG vaccination. [file 12943_2015_307_MOESM3_ESM.tiff]
